# Effectiveness of Chinese herbal medicine in treating liver fibrosis: a systematic review and meta-analysis of randomized controlled trials

**DOI:** 10.1186/1749-8546-7-5

**Published:** 2012-02-29

**Authors:** Fan Cheung, Yibin Feng, Ning Wang, Man-Fung Yuen, Yao Tong, Vivian Taam Wong

**Affiliations:** 1 School of Chinese Medicine, LKS Faculty of Medicine, The University of Hong Kong, Hong Kong SAR, China; 2 Department of Medicine, LKS Faculty of Medicine, The University of Hong Kong, Queen Mary Hospital, Pokfulam Road, Hong Kong SAR, China

## Abstract

**Background:**

The studies on the effectiveness of Chinese herbal medicines (CHM) in treating liver fibrosis (LF) were not consistent. This study aims to systematically review the effectiveness of CHM on treating LF patients.

**Methods:**

Databases including MEDLINE, AMED, EMBASE, The Cochrane Central Register of Controlled Trials, China National Knowledge Infrastructure, TCMOnline, Chinese Biomedical Literature Database, and Chinese Medical Current Contents were searched up to March 2011. Randomized controlled trials (RCTs) involving LF patients receiving CHM, Western medicine, combined CHM and Western medicine compared with placebo, Western medicine or no intervention were included. LF markers including serum hyaluronic acid (HA), laminin (LN), procollagen type III (PC-III), type IV collagen (IV-C), matrix metalloproteinase (MMP), and tissue inhibitors of metalloproteinase (TIMP) were measured as primary outcomes. Liver biochemistry, including alanine aminotransferase (ALT) and aspartarte aminotransferase (AST), and improvement of related clinical symptoms were measured as secondary outcomes. Risk of bias of allocation sequence, allocation concealment, blinding, incomplete outcome data, selective outcome reporting, and other biases were assessed.

**Results:**

Twenty-three RCTs with 2123 participants were analyzed in subgroups of types of comparison and study quality. Fifteen studies were graded as good quality. CHM alone and combined with Western medicine showed significant improvements in HA, LN, PC-III and IV-C compared with Western medicine alone. However, there were no significant differences observed between CHM and placebo treatments.

**Conclusion:**

The current inconclusive results in determining the effectiveness of CHM treatment on LF, due to the poor methodological quality and high heterogeneity of the studies, suggests that large RCTs using standardized Chinese medicine syndrome diagnosis and CHM formulae with longer follow-up are required for further evaluation.

## Introduction

Liver fibrosis (LF), as a result of wound-healing response to recurrent liver injury, is thought to be an early reversible stage of liver cirrhosis [1]. It is characterized by the formation of fibrotic scar tissue with abnormal accumulation of fibroblasts and myofibroblasts, and excessive synthesis and deposition of extracellular matrix (ECM) proteins. The development of anti-fibrotic therapy is important for patients with chronic liver diseases, especially for chronic hepatitis B (HBV) and C virus (HCV) infections [2], which are the most prevalent blood-borne viral infection and the major causes of LF worldwide, especially in mainland China [3-5]. Few LF treatments are effective and inexpensive without adverse side effect [6-8].

Categories of current research into Chinese herbal medicine (CHM) treatment of LF include (1) the prevention of anti-fibrosis effects, (2) mechanisms, and (3) clinical efficacy, safety and quality control [3]. Clinically, several studies reported the efficacy of CHM on LF [9-11]. A previous systematic review of 11 studies on LF suggested that "*Fuzheng Huayu Capsule*" had beneficial effects on LF [12]. However, the review included only one CHM compound, and the results might not be representative of all CHM. Moreover, with advanced progress on LF in recent years, the review should be updated to include recent studies.

This article aims to systematically review the published randomized controlled trials (RCTs) for evaluating the effectiveness of CHM on LF treatment.

## Methods

This study was conducted according to the Cochrane practice [13,14], including pre-specified objectives, search strategy, inclusion criteria, quality assessment, data collection and meta-analysis.

### Search strategy

Published RCTs on CHM treating LF patients were searched *via *the following electronic databases from their inception to March 2011: MEDLINE since 1948, AMED since 1985, EMBASE since 1974, and The Cochrane Central Register of Controlled Trials since 1996. In addition, four Chinese electronic databases including China National Knowledge Infrastructure (CNKI), TCMOnline, Chinese Biomedical Literature Database (CBM), and Chinese Medical Current Contents (CCMC) were searched since January 2000. The bibliographies of studies identified in the systematic search were checked for potentially relevant publications. Unpublished data were not included.

The keywords for database search were ('liver fibrosis' OR 'hepatic fibrosis' OR 'fibrotic liver' OR 'antifibrotic') AND ('Chinese medicine' OR 'traditional medicine' OR 'herbal medicine' OR 'complementary medicine' OR 'complementary therapy' OR 'alternative medicine' OR '*Fuzheng huayu*' OR 'compound 861' OR '*Anluohuaxian pill*' OR '*Rhubarb zhechong wan*' OR '*Sho saiko *to' OR '*Fufang biejiaruangan tablet*' OR '*Biejia ruanjian*' OR '*Biejiajian pill*' OR '*Qianggan capsule*' OR '*Qianggan pill*' OR '*Han-Dan-Bi-Tuo*' OR 'Matrine capsule' OR 'Oxymatrine capsule' ) AND 'randomized controlled trial' [15]. No restrictions on publication type and language of publication were imposed.

### Study selection

#### Types of studies

This review included only RCTs on the effectiveness of CHM. The studies with quasi-randomized and non-randomized study design were excluded.

#### Participants

The studies recruited patients suffering from chronic hepatitis diseases, fatty liver or *schistosomiasis japonica*, and having histologically significant LF were included. The studies involved patients having co-infection of two or more types of hepatitis or fatty liver with other chronic liver diseases, or having decompensated liver diseases were excluded.

#### Interventions

The studies comparing CHM (such as pills, tablets, capsules, decoctions, and injections) with placebo, Western medicine, or no intervention were included. CHM intervention could be a sole anti-fibrotic therapy or an adjunct treatment. The studies assessing combined effects of CHM with other intervention (*e.g*. CHM plus acupuncture, injection of CHM into acupoint, and acupoint application) were excluded. The studies used non-conventional herbal medicines or complementary medicines as control groups were also excluded. Co-intervention, including those supplements such as vitamins, was allowed if both arms of the randomized allocation received the same co-intervention.

#### Outcome measures

Primary outcome measures were mean differences (MD) of LF biomarkers, which indicate ECM metabolism, including serum hyaluronic acid (HA), laminin (LN), procollagen type III (PC-III), type IV collagen (IV-C), matrix metalloproteinase (MMP), and tissue inhibitors of metalloproteinase (TIMP) [16]. Secondary outcomes included liver biochemistry which including alanine aminotransferase (ALT) and aspartarte aminotransferase (AST), and the improvement of related clinical symptoms was defined as the alleviation of subjective symptoms after the interventions. Both non-serious and serious adverse events were evaluated. A serious adverse event included event of death, life-threatening incidents, or inpatient or prolonged of hospitalization which resulted in a persistent or significant disability [17].

### Data extraction and assessment of methodological quality

Two authors (FC and NW) independently assessed studies for eligibility, extracted data in duplicate using a structured data extraction form, and cross-checked for transcription errors. The data extraction form comprised the items of primary author, study citation, study design, participants, interventions, outcome measures, and adverse events according to pre-specific selection criteria. In case duplicate publications were found, only the most informative and updated version was included. The quality of included studies was evaluated independently by the two authors (FC and NW) using a tool for evaluating 'risk of bias' which tool was adapted from the Cochrane Handbook for Systematic Review of Interventions [13] with slight modifications for transforming the bias codes of "yes", "unclear" and "no" into 2, 1 and 0, respectively, for presenting clearer results. The following six questions were asked:

(1) Was the allocation sequence adequately generated?

(2) Was allocation adequately concealed?

(3) Was knowledge of the allocated interventions adequately prevented during the study?

(4) Were incomplete outcome data adequately addressed?

(5) Are reports of the study free of suggestion of selective outcome reporting?

(6) Was the study free of other problems that could put it at a risk of bias?

Every item would be given 2 points for answering 'yes', 1 for answering 'unclear' and 0 for answering 'no'. Prevention of knowledge of the allocated interventions, i.e. blinding (patient, personnel, and outcome assessor blinding), was assessed separately. There were eight items in total as three items from blinding (including blinding of patients, personnel, and outcome assessor), and five from the rest five questions. The scale ranged from 0 to 16 points with 0 to 7 regarded as poor quality, and 8 or above as good quality. Any disagreement was resolved by consensus. If necessary, the third author (YF) was consulted for resolution.

### Data synthesis of outcome measures

Review Manager, Version 5.1 for Windows (The Nordic Cochrane Centre, Copenhagen, Denmark) and STATA 10.1 (StataCorp, College Station, TX, USA) were used for data analysis. Meta-analysis was carried out on the intention-to-treat (ITT) basis regardless whether the subjects were lost to follow-up. Subgroup analysis was conducted among different comparisons (including CHM *versus *Western medicine, CHM versus placebo, and combined treatment *versus *Western medicine) and study quality (all studies *versus *the studies with good quality). Inverse-variance random effects model was used MD and 95% confidence intervals (CI) were calculated. Heterogeneity was assessed by examining the forest plots and *I^2 ^*statistics, where *I^2 ^*values of 25%, 50%, and 75% were regarded as low, moderate, and high heterogeneity, respectively [18]. Sensitivity analysis for primary outcomes was performed to assess the impact of excluding outlier studies when there was high heterogeneity (*I^2 ^*> 75%) between studies. In a three-arm study that had two control groups of conventional medicine and no intervention, the treatment group was split into two groups to create two comparisons in the meta-analysis. Funnel plot and Egger's regression asymmetry test were applied to detect for the potential publication bias [19]. *P *value less than 0.05 were considered statistically significant.

## Results

### Study characteristics

**Figure 1:**
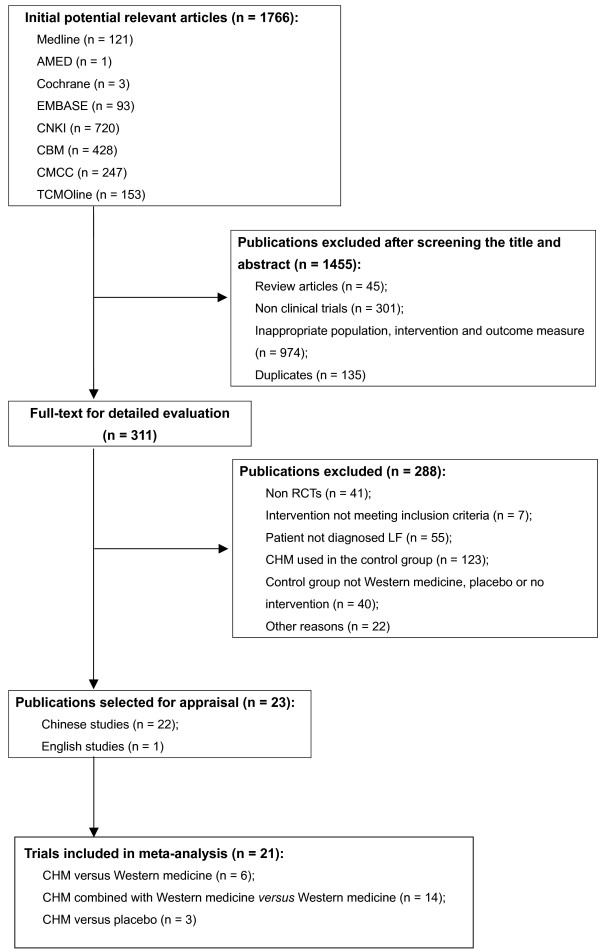
**Flow diagram of study selection process**.

Figure 1 shows the process of the study selection. Twenty-three RCTs with a total of 2123 participants met the eligibility criteria were included in this review (Table 1). Sample sizes ranged from 44 to 164. Among these included studies, a study recruited *schistosomiasis japonica *patients only [20], the remaining recruited patients with chronic HBV infection. All of the identified studies were conducted in China and published in Chinese language, and only one study was published in English language [21]. Three studies used three-arm study design (2 intervention groups compared with 1 control group, or 1 intervention group compared with 2 control groups) [21-23] and others used two-arm study design (1 intervention group versus 1 control group) [20,24-42]. No study reported mortality, liver cirrhosis or cancer, quality of life or cost as outcomes. The information about the study design, participants, intervention, outcome assessment, and quality was presented in Additional file 1.

**Table 1:** Characteristics of the included studies

Study ID	Sample size	Intervention Group	Control Group	Duration Primary Outcomes		Secondary Outcomes
Chen 2005 [24]	49	'Fufang Biejia Ruangan Tablets' + IFN-γ (n = 28)	IFN-γ (n = 21)	6 months	HA, LN, PC-III, IV-C & Knodell score	ALT & AST
Chen 2006 [33]	116	Kang Xian Decoction + IFN-α (n = 58)	IFN-α (n = 58)	6 months	HA, LN & PC-III	ALT & Improvement of related clinical symptoms
Chen 2006_2 [20]	138	'Qianggan Capsule' + conventional care (n = 68)	Placebo + conventional care (n = 70)	6 months	HA, LN, PC-III & IV-C	ALT, Improvement of related clinical symptoms
Chen 2007 [22]	164	Group I: Bie Jia Jian Pills + conventional care (n = 54)	IFN + conventional care (n = 52)	9 months	HA, LN & PC-III	
		Group II: Group I + control group (n = 58)				
Chen 2010 [25]	96	'Fufang Biejia Ruangan Tablets' + Entecavir (n = 46)	Entecavir (n = 50)	1 year	HA, LN, PC-III & IV-C	ALT & AST.
Dai 2011 [26]	68	'Fufang Biejia Ruangan Tablets' + Entecavir (n = 34)	Entecavir (n = 34)	1 year	HA, LN, PC-III & IV-C	ALT, AST & Improvement of related clinical symptoms
Gao 2000 [34]	120	'HB-Granule-3' (n = 60)	IFN-α (n = 60)	90 days	HA, LN & IV-C	ALT & Improvement of related clinical symptoms
Huang 2007 [27]	99	'Decoction of Radix Salviae Milltorrhizae, Radix Astragali and Rhubarb' (n = 50)	IFN-α (n = 49)	3 months	HA, LN, PC-III & IV-C	ALT & AST
Huang 2009 [28]	83	'Fufang Biejia Ruangan Tablets' + Adefovir dipivoxil (n = 43)	Adefovir dipivoxil (n = 40)	1 year		ALT & AST
Kuang 2005 [29]	53	Bie Jia Jian Decoction (n = 27)	LVD (n = 26)	60 days	HA, PC-III & IV-C	ALT, AST & Improvement of related clinical symptoms
Li 2006 [30]	60	'Xiexian Oral Liquid' + conventional care (n = 30)	Placebo + conventional care (n = 30)	4 months	HA, LN & IV-C	ALT, AST & Improvement of related clinical symptoms
Li 2011 [31]	88	'Anluo Huaxian Pills' + Adefovir dipivoxil (n = 44)	Adefovir dipivoxil (n = 44)	9 months	HA, LN, PC-III & IV-C	ALT & AST
Lu 2010 [32]	82	'Fufang Biejia Ruangan Tablets' + Adefovir dipivoxil (n = 42)	Adefovir dipivoxil (n = 40)	1 year	HA, LN, PC-III & IV-C	ALT, AST & Improvement of related clinical symptoms
						
Shen 2003 [35]	68	'Ganxian Prescirption' + LVD (n = 31)	LVD (n = 37)	1 year	HA, LN & IV-C	ALT & AST
Shen 2005 [21]	120	Group I: 'Ganxian Recipe' (n = 40)	LVD (n = 40)	2 years	HA, LN & IV-C	ALT & AST
		Group II: 'Ganxian Recipe' + LVD				
		(n = 40)				
Sun 2010 [36]	55	'Anluo Huaxian Pills' + Adefovir dipivoxil (n = 30)	Adefovir dipivoxil (n = 25)	48 weeks	HA, LN, PC-III & IV-C	ALT, AST & Improvement of related clinical symptoms.
						
Wang 2006 [23]	160	Group I: experienced clinical decoction (n = 50)	LVD (n = 50)	6 months	HA, LN, PC-III & IV-C	
		Group II: Group I + Control Group (n = 60)				
Wang 2010 [37]	98	'Fufang Biejia Ruangan Tablets' + Adefovir dipivoxil (n = 49)	Adefovir dipivoxil (n = 49)	1 year	HA, LN, PC-III & IV-C	ALT
Wei 2010 [38]	44	'Fufang Biejia Ruangan Tablets' + Adefovir dipivoxil (n = 22)	Adefovir dipivoxil (n = 22)	1 year	HA, LN, PC-III & IV-C	ALT & AST
Xie 2009 [39]	62	'Huaxian Fugan Prescription' + conventional care (n = 32)	Conventional care (n = 30)	6 months	HA, LN, PC-III & IV-C	ALT, AST & Improvement of related clinical symptoms
Yang 2009 [40]	120	'Fufang Biejia Ruangan Tablets' + LVD + conventional care (n = 60)	LVD + conventional care (n = 60)	6 months	HA, LN, PC-III & IV-C	ALT & AST
Yin 2004 [41]	102	'Herbal Compound 861' (n = 52)	Placebo (n = 50)	24 weeks	HA, LN, PC-III, IV-C, MMPI, MMP2, MMP9, TIMPI & TIMP2	ALT, AST & Improvement of related clinical symptoms
Zhang 2000 [42]	78	'Kanggan Xianfang' + conventional care (n = 39)	Conventional care (n = 39)	6 months	HA, PC-III & TGF-β1	ALT & Improvement of related clinical symptoms

### Treatment groups

The types of intervention were classified as CHM (N = 8) and combined treatment (CHM plus Western medicine) (N = 15) including Interferon (IFN), Entecavir, Adefovir Dipivoxil, and Lamivudine (LVD). CHM was prepared as decoctions (N = 7), tablets (N = 8), granules (N = 2), capsules (N = 2), pills (N = 3), and oral liquid (N = 1). Only standardized (87%, 20/23) and semi-standardized (13%, 3/23) CHM prescriptions were used in these studies. The standardized prescriptions indicate fix formulas for all participants and the semi-standardized prescriptions were defined as individually customized formulas according to Chinese medicine.

### Control groups

Comparison groups included Western medicines (including IFN, Entecavir, Adefovir Dipivoxil, and LVD), placebo, and no intervention. Three studies used placebos, with one using physiological saline and food coloring [30], and two using similar shape capsules [20,41].

### Duration of follow-up

The range of intervention duration in the studies ranged from 60 days to two years with mostly were six months (N = 7) and one year (N = 7). The duration of follow-up was only reported in three studies with a range from three to six months [27,30,34].

### Methodological quality

Fifteen studies were graded as good quality and others as poor quality (as shown in Additional file 1). Out of 23 included studies, only two studies reported adequate generation of allocation sequence using random number tables or drawing of lots for assigning groups [29,41]. None of the studies described the method of allocation concealment. Only one study reported using blinding design (single blind without description of the blinding method) [21] and three studies used placebos [20,30,41]. Six studies did not provide the information on missing data [28,29,33-35,41]. None reported the use of ITT in their analysis.

### Outcomes

#### Primary outcomes

**Figure 2:**
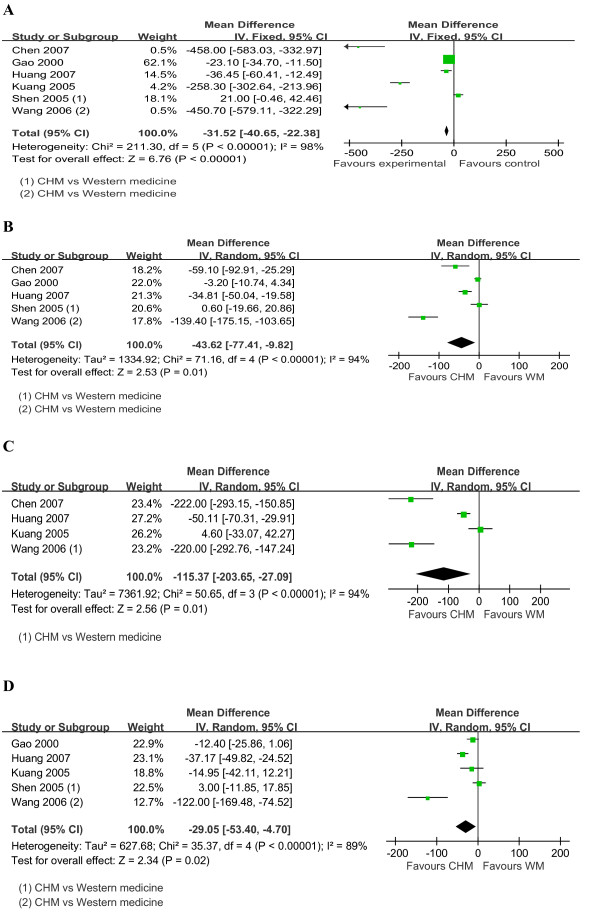
**Forest plot of studies comparing Chinese herbal medicine and western medicine, examining the effect on liver fibrosis markers (including HA, LN, PC-III and IV-C)**. **(A) **HA. **(B) **LN. **(C) **PC-III. **(D) **IV-C. Vertical line represents no effect point; CI, confidence interval; HA, hyaluronic acid; LN, laminin; PC-III, procollagen type III; IV-C, type IV collagen.

**Figure 3:**
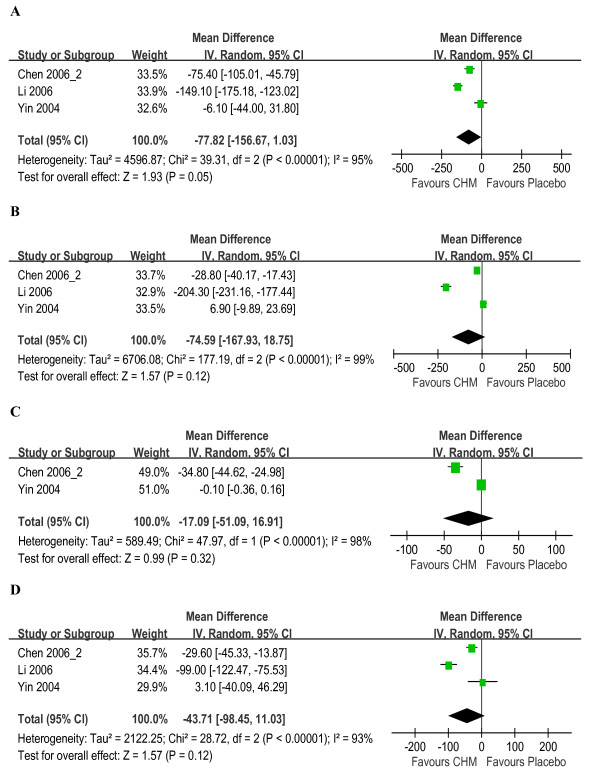
**Forest plot of studies comparing Chinese herbal medicine and placebo medicine, examining the effect on liver fibrosis markers (including HA, LN, PC-III and IV-C)**. **(A) **HA. **(B) **LN. **(C) **PC-III. **(D) **IV-C.

**Figure 4:**
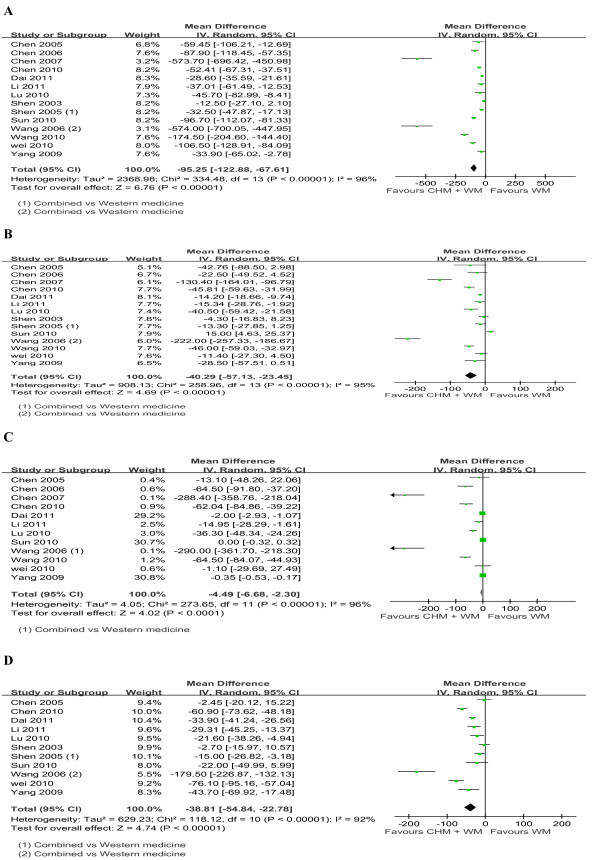
**Forest plot of studies comparing combined medicine and western medicine, examining the effect on liver fibrosis markers (including HA, LN, PC-III and IV-C)**. (A). HA. **(B) **LN. **(C) **PC-III. **(D) **IV-C.

Figures 2, 3, 4 show the forest plots of MD of LF markers (HA, LN, PC-III & IV-C) with 95% CI.

CHM group *versus *Western medicine group (6 studies):

CHM significantly reduced the levels of HA (pooled MD-31.52; 95% CI-40.65, -22.38; *P *< 0.00001), LN (pooled MD-43.62; -77.41, -9.82; *P *= 0.01), PC-III (pooled MD-115.37; -203.65, -27.09; *P *= 0.01) and IV-C (pooled MD-29.05; -53.4, -4.7; *P *= 0.02), with *I^2 ^*ranging from 89% to 98%, as shown in Figure 2. Subgroup analyses among studies with different quality also found substantial significant differences in favor of CHM in the levels of HA (pooled MD -292.65; 95% CI-481.77, -103.54; *P *= 0.002), LN (pooled MD-76.28; -136.23, -16.32; *P *= 0.01), PC-III (pooled MD-115.37; -203.65, -27.09; *P = *0.01) and IV-C (pooled MD -52.05; -93.95, -10.15; *P *= 0.01), with *I^2 ^*ranging from 86% to 98%. The significance differences were maintained for the levels of HA (pooled MD - 21.46; 95% CI -30.80, - 12.13; *P *< 0.00001), LN (pooled MD -21.01; 95% CI -43.49, 1.47; *P *= 0.07), and IV-C (pooled MD -15.65; 95% CI -34.57, 3.26; *P *= 0.1) when excluding the studies with outlier results, with *I^2 ^*ranging from 83% to 96%. For PC-III (pooled MD -221.02; 95% CI -271.89, -170.15; *P *< 0.00001), the heterogeneity was largely reduced after removing two potential outlier studies [27,29] (overall *I^2 ^*= 0%).

CHM group *versus *placebo group (3 studies):

CHM had no significant effects on the levels of HA (pooled MD -77.82; 95% CI-156.67, 1.03; *P *= 0.05), LN (pooled MD -74.59; -167.93, 18.75; *P *= 0.12), PC-III (pooled MD -17.09; -51.09, 16.91; *P *= 0.32) and IV-C (pooled MD -43.71; -98.45, 11.03; *P *= 0.12) when compared with placebo, with *I^2 ^*ranging from 93% to 99%, as shown in Figure 3. Subgroup analysis showed the same estimates for quality and sensitivity analyses excluding outliers found similar results for the levels of HA (pooled MD -41.80; 95% CI-109.68, 26.08; *P *= 0.23), LN (pooled MD -11.51; 95% CI -46.48, 23.46; *P *= 0.52), and IV-C (pooled MD -19.69; 95% CI-49.15, 9.76; *P *= 0.19) A large high heterogeneity was observed (*I^2 ^*ranging from 87% to 92%) except in IV-C (*I^2 ^*= 49%).

Combined treatment group *versus *Western medicine group (14 studies):

Combined treatment was found to significantly reduce the levels of HA (pooled MD -46.59; 95% CI -51.23, -41.944; *P *< 0.00001), LN (pooled MD -40.292; -57.13, -23.45; *P *< 0.00001), PC-III (pooled MD -4.49; -6.68, -2.3; *P *< 0.0001) and IV-C (pooled MD -38.81; -54.84, -22.78; *P *< 00001) compared with Western medicine, Western medicine, with *I^2 ^*ranging from 92% to 96%, as shown in Figure 4. Subgroup analyses showed high significant differences for good quality studies in the levels of HA (pooled MD -121.46; 95% CI-166.40, -76.51; *P *< 0.00001), LN (pooled MD -58.53; -88.38, -28.68; *P *= 0.0001), PC-III (pooled MD) - 13.14; -18.81, -7.48; *P *< 0.00001), and IV-C (pooled MD -44.45; -68.17, -20.73; *P *= 0.0002), with *I^2 ^*ranging from 93% to 97%. Sensitivity analyses excluding outliers found similar estimated and heterogeneity in the levels of HA (pooled MD -41.94; 95% CI-46.65, -37.23; *P *< 0.00001), LN (pooled MD -24.22; -33.77, -14.67; *P *< 0.00001), PC-III (pooled MD -2.57; -4.20, -0.94; *P *= 0.002) and IV-C (pooled MD -30.53; -44.35, -16.70; *P *< 0.0001), with *I^2 ^*ranging from 80% to 94%.

#### Secondary outcomes

Comparing with Western medicine, combined treatment was statistically significant in reducing ALT level (pooled MD -11.35; 95% CI -18.75, -3.95; *I^2 ^*= 85%; *P *= 0.003). No significant difference was found in AST level (pooled MD -1.13; 95% CI -6.56, 4.3; *I^2 ^*= 52%; *P *= 0.68). However, when comparing CHM with Western medicine and placebo, no significant difference was found for the levels of ALT (For CHM *versus *Western medicine: pooled MD -14.59; 95% CI -37.190, 8; *I^2 ^*= 95%; *P *= 0.21. For CHM *versus *placebo: pooled MD -18.64; -52.89, 15.61; *I^2 ^*= 73%; *P *= 0.29) and AST (For CHM *versus *Western medicine: pooled MD 9.7; -3.37, 22.76; *I^2 ^*= 88%; *P *= 0.15. For CHM *versus *placebo: pooled MD -17.94; -37.57, 1.69; *I^2 ^*= 37%; *P *= 0.07).

Eleven studies [20,26,29,30,32-34,36,39,41,42] reported symptom improvement with eight studies [20,29,30,32,33,36,39,42] reported statistically significant difference (*P *< 0.05) in the outcomes comparing CHM with Western medicine, placebo, and no intervention; and comparing combined treatment with Western medicine. One out of the eleven studies comparing CHM with placebo reported a non-significant improvement [41].

#### Adverse events

Although adverse events were reported in nine studies, none was serious. Among these, three reported no adverse event in both groups [31,32,35] while one reported no adverse event for CHM [30]. Five reported gastrointestinal discomfort and drug-allergic symptoms for CHM, placebo, IFN and LVD [20,21,27,30,41]. One reported two cases with dizziness using the combined treatment [21]. Other adverse symptoms of flu-like symptoms or mild leucopenia and thrombocytopenia were related to the use of IFN or LVD [21,22,27]. No study described the method of data collection for adverse events.

#### Evaluation of publication bias

Publication bias was found for HA (*P *= 0.003), PC-III (*P *= 0.001) and LN (*P *= 0.047) although non-significant for IV-C (*P *= 0.814) according to Egger's test. However, visual inspection of the funnel plots (please see funnel plots in Additional file 2) found no obvious basis.

## Discussion

### Overall findings

The levels of LF markers were significantly reduced in patients receiving CHM or combined treatment compared with Western medicine. The levels were significantly decreased in CHM group compared with no intervention although the effect was not significantly different when in comparison with Western medicine or placebo. Moreover, CHM was found to be effective in symptom improvements. It should be noted that CHM was not consistently better than placebo.

### Methodological quality of studies

Eight studies [21,25,28,31,33,34,36,37] were assessed to be poor quality according to modified Cochrane 'risk of bias' scale. Only two studies reported the method of randomization [34,41]. The method of allocation concealment was not reported by all studies, which should be alert to the possibility of selection bias and overestimation of intervention effects [43]. Blinding was not reported or inappropriately reported by most of the studies. Although most studies used objective outcome measures, it did not rule out the possibility of performance bias and detection bias [44].

### Potential biases

Strict eligibility criteria were used to reduce heterogeneity. The included studies, however, had various participants' characteristics and different CHM or combined treatment against different control interventions. Small sample sizes, methodological differences between studies and variations in study objectives might contribute to heterogeneity. To investigate the high levels of heterogeneity in this meta-analysis we performed subgroup analysis and sensitivity analysis. These analyses did not find inconsistency.

Some high quality studies might be missed due to the strict eligibility criteria such as a multicentre, double blinded RCT comparing "*Fuzhenghuayu capsule*" with "*Heluoshugan capsule*" [45]. Other reviews including CHM as control may be needed in the future. As most studies were of small scale and poor methodological quality, large RCTs of high quality would be required for determining the effectiveness of CHM on LF treatment.

In order to minimize bias in the review, we did not restrict the publication type and language, and searched many commonly accessed databases. However, all identified studies were conducted in China, and studies more likely reported positive results, which may be influenced by publication and location bias [46-48]. Our analysis of publication bias using Egger's test did show publication bias in the outcomes of HA, LN, and PC-III, although the funnel plots were symmetric in distribution (Additional file 2).

### Limitations and further research

Most of the current studies only focused on the effectiveness of intervention [49] but neglected monitoring the harmful effect from CHM. Further studies should assess both the safety and effectiveness. Well-designed, multi-centre and large sample size RCTs in compliance with the CONSORT guideline [50] should be implemented. Studies with CHM should be registered before their conduct.

## Conclusion

The current inconclusive studies are of poor methodological quality and high heterogeneity do not adequately support the effectiveness of CHM treatment on LF. Large RCTs using standardized Chinese medicine syndrome diagnosis and CHM formulae with longer follow-up are required for further evaluation.

## Abbreviations

ALT: Alanine aminotransferase; AST: Aspartarte aminotransferase; CBM: Chinese Biomedical Literature Database; CCMC: Chinese Medicine Current Content; CHM: Chinese herbal medicine; CI: Confidence interval; CNKI: China National Knowledge Infrastructure; ECM: Extracellular matrix; HA: Hyaluronic acid; HBV: Hepatitis B virus; HCV: Hepatitis C virus; IFN: Interferon; ITT: Intention-to-treat analysis; IV-C: Type IV collagen; LF: Liver fibrosis; LN: Laminin; LVD: Lamivudine; MD: Mean difference; MMP: Matrix metalloproteinase; PC-III: Procollagen type III; RCTs: Randomized controlled trials; TIMP: Tissue inhibitors of metalloproteinase.

## Competing interests

The authors declare that they have no competing interests.

## Authors' contributions

FC conducted the database search, assessed studies for inclusion, extracted and analyzed the data, and drafted the manuscript. YF conceived the study, analyzed the data, and revised the manuscript. NW conducted the database search, assessed studies for inclusion, extracted the data which followed by cross checking with FC, analyzed the data, and drafted the manuscript. MFY, YT and VTW interpreted the data and revised the manuscript. All authors read and approved the final version of the manuscript.

## Supplementary Material

Additional file 1 **Details of characteristics and methodological quality of the included studies**. Click here for file

Additional file 2 **Funnel plots (with pseudo 95% CI) for the primary outcomes (including HA, LN, PC-III and IV-C) of the included studies in the meta-analysis**. (A) HA. (B) LN. (C) PC-III. (D) IV-C. Click here for file

## References

[B1] Bataller R Brenner DA Liver fibrosis J Clin Invest 2005 115 2 209 218 15690074 10.1172/JCI24282 PMC546435

[B2] Ramachandran P Iredale JP Reversibility of liver fibrosis Ann Hepatol 2009 8 4 283 291 20009126

[B3] Feng Y Cheung KF Wang N Liu P Nagamatsu T Tong Y Chinese medicines as a resource for liver fibrosis treatment Chin Med 2009 4 16 10.1186/1749-8546-4-16 19695098 PMC3224967

[B4] Nie QH Zhu CL Zhang YF Yang J Zhang JC Gao RT Inhibitory effect of antisense oligonucleotide targeting TIMP-2 on immune-induced liver fibrosis Dig Dis Sci 2010 55 5 1286 1295 10.1007/s10620-009-0858-5 19517234

[B5] Liu J Fan D Hepatitis B in China Lancet 2007 369 9573 1582 1583 10.1016/S0140-6736(07)60723-5 17499584

[B6] Lai CL Ratziu V Yuen MF Poynard T Viral hepatitis B Lancet 2003 362 9401 2089 2094 10.1016/S0140-6736(03)15108-2 14697813

[B7] Myers RP Regimbeau C Thevenot T Leroy V Mathurin P Opolon P Zarski JP Poynard T Interferon for interferon naive patients with chronic hepatitis C Cochrane Database Syst Rev 2002 2 CD000370 12076394 10.1002/14651858.CD000370 PMC7061493

[B8] Rambaldi A Gluud C Colchicine for alcoholic and non-alcoholic liver fibrosis and cirrhosis Cochrane Database Syst Rev 2005 2 CD002148 15846629 10.1002/14651858.CD002148.pub2 PMC8437891

[B9] Liu CH Hu YY Xu LM Liu C Liu P Effect of Fuzheng Huayu formula and its actions against liver fibrosis Chin Med 2009 4 12 10.1186/1749-8546-4-12 19558726 PMC2720970

[B10] Huang JD The clinical curative effect of Jianpirougan Decoction in the treatment of chronic hepatitis b liver fibrosis Zhong Yi Xue Bao 2010 25 6 1175 1177

[B11] Yang H Chen Y Xu R Shen W Chen G Clinical observation on the long-term therapeutic effects of traditional Chinese medicine for treatment of liver fibrosis J Tradit Chin Med 2000 20 4 247 250 11263273

[B12] Li L He Q Yang DG Zhong BL Zeng XM Effectiveness and safety of fuzheng huayu capsule for liver fibrosis of chronic hepatitis b: A systematic review Zhongguo Xun Zheng Yi Xue 2006 8 10 892 897

[B13] Higgins JPT, Green S Cochrane Handbook for Systematic Reviews of Interventions Version 5.1.0 [updated March 2011] The Cochrane Collaboration 2011

[B14] Khan KS Dinnes J Kleijnen J Systematic reviews to evaluate diagnostic tests Eur J Obstet Gynecol Reprod Biol 2001 95 1 6 11 10.1016/S0301-2115(00)00463-2 11267714

[B15] Liver Disease Committee Chinese Association of Integrative Medicine Guideline for the diagnosis and treatment of liver fibrosis with integrative medicine Zhongguo Zhong Xi Yi Jie He Za Zhi 2006 26 11 1052 1056 17186743

[B16] Zois CD Baltayiannis GH Karayiannis P Tsianos EV Systematic review: hepatic fibrosis-regression with therapy Aliment Pharmacol Ther 2008 28 10 1175 1187 10.1111/j.1365-2036.2008.03840.x 18761707

[B17] International Conference on Harmonisation Code of Federal Regulations & ICH Guidelines 1997 Philadelphia, US: Barnett International/PAREXEL22390424

[B18] Higgins JP Thompson SG Deeks JJ Altman DG Measuring inconsistency in meta-analyses BMJ 2003 327 7414 557 560 10.1136/bmj.327.7414.557 12958120 PMC192859

[B19] Egger M Smith GD Schneider M Minder C Bias in meta-analysis detected by a simple, graphical test BMJ 1997 315 7109 629 634 10.1136/bmj.315.7109.629 9310563 PMC2127453

[B20] Chen SX Hou XY Li Y Clinic study on Qianggan capsule for fibrosis of liver in early stage due to schistosomiasis japonica Re Dai Bing Yu Ji Sheng Chong Xue 2006 4 2 78 80

[B21] Shen WS Yang HZ Hong Q Zhang YQ Xie HP Bian Z Two-year observation of the clinical efficacy in treating chronic hepatitis B patients with Ganxian Recipe and lamivudine Chin J Integr Med 2005 11 1 5 10 10.1007/BF02835740 15975299

[B22] Chen LH Chen HQ Chen W Hong GM Combined use of Bie Jia Jian Pills and interferon in managing chronic hepatitis B fibrosis Xian Dai Zhong Xi Yi Jie He Za Zhi 2007 16 33 4931 4932

[B23] Wang DF 60 cases of treating chronic hepatitis B fibrosis by combining Chinese medicine with western medicine Zhongguo Zhong Xi Yi Jie He Za Zhi 2006 16 5 294 296

[B24] Chen LJ Ye SF Clinical Research of Fufangbiejiaruangan tablet combined with γ-interferon on treating hepatic fibrosis Zhejiang Zhong Xi Yi Jie He Za Zhi 2005 15 10 595 596

[B25] Chen CR Guo JC Yu XL Wang YF Entecavir Combined with Compound Biejia Ruangan Tablets Treat Chronic hepatitis b fibrosis Zhejiang Zhong Yi Yao Da Xue Xue Bao 2010 34 3 370 371

[B26] Dai WW Feng YH Chang JB Qiu J The clinical observation of treatment of hepatitis b cirrhosis using entecavir combined Fufang Biejia Ruangan tablets Gansu Zhong Yi 2011 24 2 28 29

[B27] Huang TX Wu SK Song SL Li J Zhu Y Clinical study on compound decoction of Radix Salviae Milltorrhizae, Radix Astragali and Rhubarh against hepatic fibrosis in chronic hepatitis b patients Lin Chuang Jun Yi Za Zhi 2007 35 3 361 364

[B28] Huang WL Liang LZ Clinical observation of adefovir combined Fufang Biejie Ruangan tablets treating early stage hepatitis b cirrhosis Zhongguo Shi Yong Yi Yao 2009 4 7 158 159

[B29] Kuang WH Effects of Bie Jia Jian Decoction on 27 patients with chronic hepatitis B Shandong Zhong Yi Za Zhi 2005 24 11 655 657

[B30] Li TY Li R Chen BY Xia JM Cheng J Li WZ Zheng HQ Clinical research of Xiexian oral liquid on hepatic fibrosis in chronic hepatitis Zhong Xi Yi Jie He Gan Bing Za Zhi 2006 16 4 201 203

[B31] Li GS Zhao D Clinical observation of Anluo Huaxian Pills treating liver fibrosis Zhongguo She Qu Yi Shi 2011 13 274 202 203

[B32] Lu N Xu Y Cheng WN Clinical research of adefovir dipivoxil tablets combined with Fufang Biejia Ruangan tablets on treating hepatic fibrosis following chronic hepatitis B Zhong Xi Yi Jie He Gan Bing Za Zhi 2010 20 4 209 211

[B33] Chen FQ Lin XT Peng JL A clinical study of α-interferon plus Kang Xian Decoction in treating chronic hepatitis B fibrosis and early-stage liver cirrhosis Shi Yong Yi Xue Za Zhi 2006 22 9 1063 1065

[B34] Gao H Zhou DQ Xiong YQ Zheng XY Zhou XZ Peng LS Qiu M Qi YP Zhou J Xu WJ Rao WL Zheng YJ Therapeutic effect of HB-Granule-3 on hepatic fibrosis Zhong Xi Yi Jie He Gan Bing Za Zhi 2000 6 5 6

[B35] Shen WS Yang HZ Hong Q Zhang YQ Dai M Clinical observation on the effect of Ganxian prescription combined lamivudine in treating 31 patients with chronic hepatitis B Zhongguo Zhong Xi Yi Jie He Ji Jiu Za Zhi 2003 10 5 290 292

[B36] Sun YR Li XE Clinical observation of Anluo Huaxian Pills combined adefovir dipivoxil treating chronic hepatitis b fibrosis Xian Dai Zhong Yi Yao 2010 30 5 31 32

[B37] Wang HR Clinical observation of adefovir dipivoxil combined Fufang Biejia Ruangan tablets treating chronic hepatitis b fibrosis She Qu Yi Xue Za Zhi 2010 8 3 27

[B38] Wei ML Clinical observation of adefovir dipivoxil capsules combined Fufang Biejia Ruangan tablets treating early stage Cirrhosis Shi Yong Xin Nao Fei Xue Guan Bing Za Zhi 2010 18 9 1273 1274

[B39] Xie BH Yang JM Wu YW Clinical research on Huaxianfugan prescription in treating hepatic fibrosis Tianjin Zhong Yi Yao 2009 26 2 108 109

[B40] Yang SW Zhang CH Clinical observation of lamivudine combined Fufang Biejia Ruangan tablets treating chronic hepatitis b fibrosis Yi Xue Xin Xi 2009 22 3 391

[B41] Yin SS Wang BB Wang TL Gu JD Qian LX Clinical study of compound 861 treating chronic hepatitis b fibrosis and early stage cirrhosis Zhonghua Gan Zang Bing Za Zhi 2004 12 8 467 470 15329205

[B42] Zhang YB Ceng H Wang DH Wang J Observation on curative effect of post-hepatitic fibrosis treated by Kanggan Xianfang Hubei Zhong Yi Za Zhi 2000 22 4 13 14

[B43] Pildal J Hro' bjartsson A Jørgensen K Hilden J Altman D Gøtzsche P Impact of allocation concealment on conclusions drawn from meta-analyses of randomized trials Int J Epidemiol 2007 36 847 857 10.1093/ije/dym087 17517809

[B44] Schulz KF Grimes DA Blinding in randomized trials: hiding who got what Lancet 2002 359 9307 696 700 10.1016/S0140-6736(02)07816-9 11879884

[B45] Liu P Hu YY Liu C Xu LM Liu CH Sun KW Hu DC Yin YK Zhou XQ Wan MB Cai X Zhang ZQ Ye J Zhou RX He J Tang BZ Multicentre clinical study on Fuzhenghuayu capsule against liver fibrosis due to chronic hepatitis B World J Gastroenterol 2005 11 9 2892 2899 15902724 10.3748/wjg.v11.i19.2892 PMC4305655

[B46] Egger M Smith GD Bias in location and selection of studies BMJ 1998 316 61 66 10.1136/bmj.316.7124.61 9451274 PMC2665334

[B47] Pittler MH Abbot NC Harkness EF Ernst E Location bias in controlled clinical trials of complementary/alternative therapies J Clin Epidemiol 2000 53 485 489 10.1016/S0895-4356(99)00220-6 10812320

[B48] Vickers A Goyal N Harland R Rees R Do certain countries produce only positive results? A systematic review of controlled trials Control Clin Trials 1998 19 2 159 166 10.1016/S0197-2456(97)00150-5 9551280

[B49] Barnes J Quality, efficacy and safety of complementary medicines: fashions, facts and the future. Part I. Regulation and quality Br J Clin Pharmacol 2003 55 3 226 233 10.1046/j.1365-2125.2003.01810.x 12630971 PMC1884210

[B50] CONSORT statement http://www.consort-statement.org/

