# Supplementary material for: Effectiveness of Chinese herbal medicine in treating liver fibrosis: a systematic review and meta-analysis of randomized controlled trials
Source: Chin Med. 2012 Feb 29;7:5. doi: 10.1186/1749-8546-7-5 (PMC3310806; doi:10.1186/1749-8546-7-5)
Supplement: Additional file 1 — Details of characteristics and methodological quality of the included studies. [file 1749-8546-7-5-S1.DOCX]

Additional file 1. Details of characteristics and methodological quality of the included studies.

| Study ID | Total No. of Patients  (Intervention/Control) | Intervention Group | Control Group | Duration | Primary Outcomes | Secondary Outcomes | Follow up | Quality |
| --- | --- | --- | --- | --- | --- | --- | --- | --- |
| Chen 2005 [21] | 49 (28/21) | ‘Fufang Biejia Ruangan Tablets’ (standardized prescription; oral 4 tablets, TID) and access to IFN-γ 1MU (IM). | IFN-γ 1MU (IM). | 6 months | HA: intervention group better than control group (*P* < 0.05).  NS in LN, PC-III & IV-C. | NS in ALT & AST. | None | Good  (Modified Cochrane risk of bias 9/16) |
| Chen 2006 [30] | 116 (58/58) | Kang Xian Decoction (standardized prescription; oral, one dose per day) and access to IFN-α 3MU. | IFN-α 3MU. | 6 months | HA, LN & PC-III: intervention group better than control group ( *P* < 0.05). | ALT & improvement of related clinical symptoms: intervention group better than control group (*P* < 0.01). | None | Poor  (Modified Cochrane risk of bias 5/16) |
| Chen 2006_2 [20] | 138 (68/70) | ‘Qianggan Capsule’ (standardized prescription; oral, 4 g/d) plus conventional care. | Placebo (shape consistent with intervention group) plus conventional care. | 6 months | HA, LN, PC-III & IV-C: intervention group better than control group ( *P* < 0.05). | ALT, improvement of related clinical symptoms: intervention group better than control group (*P* < 0.05).  Adverse events: 4 cases in intervention group and 5 in control group- nausea, vomiting and abdominal discomfort. | None | Good  (Modified Cochrane risk of bias 10/16) |
| Chen 2007 [31] | 164 (54/58/52) | Group I: Bie Jia Jian Pills (standardized prescription; oral, 6 g/d) plus conventional care.  Group II: Group I plus control group. | IFN 3MU (IM) plus conventional care. | 9 months | HA: intervention group better than control group at 6 & 9 months (*P* < 0.01).  LN & PC-III: Group II better than control group at 6 & 9 months (*P* < 0.01), NS compared Group I with control group. | Adverse events: In the use of IFN therapy, 80% patients had cold or “flu-like” symptoms; some patients with mild leucopenia and thrombocytopenia. | None | Good  (Modified Cochrane risk of bias 10/16) |
| Chen 2010 [22] | 96 (46/50) | ‘Fufang Biejia Ruangan Tablets’ (standardized prescription; oral 2 g, TID) and access to Entecavir 0.5 mg (oral, QD). | Entecavir 0.5 mg (oral, QD). | 1 year | HA, LN, PC-III & IV-C: intervention group better than control group (*P* < 0.05). | NS in ALT & AST. | None | Poor  (Modified Cochrane risk of bias 7/16) |
| Dai 2011 [23] | 68 (34/34) | ‘Fufang Biejia Ruangan Tablets’ (standardized prescription; oral 2 g, TID) and access to Entecavir 0.5 mg (oral, QD). | Entecavir 0.5 mg (oral, QD). | 1 year | HA, LN, PC-III & IV-C: intervention group better than control group (*P* < 0.05). | NS in ALT & AST.  Improvement of related clinical symptoms: intervention group better than control group (just description). | None | Good  (Modified Cochrane risk of bias 8/16) |
| Gao 2000 [32] | 120 (60/60) | ‘HB-Granule-3’ (standardized prescription; oral 10 g, TID). | IFN-α 3MU (SC, three times per week). | 90 days | HA & IV-C: intervention group better than control group (p<0.05).  NS in LN. | ALT (*P* < 0.01) & improvement of related clinical symptoms: intervention group better than control group (just description). | 3 months | Poor  (Modified Cochrane risk of bias 3/16) |
| Huang 2007 [24] | 99 (50/49) | ‘Decoction of Radix Salviae Milltorrhizae, Radix Astragali and Rhubarb’ (standardized prescription; oral 100 mL, BID). | IFN-α 5MU (IM, once every two days). | 3 months | HA & PC-III: intervention group better than control group (*P* < 0.01) at 3-month, NS at 6-month follow-up.  LN & IV-C: intervention group better than control group at 3-month (*P* < 0.01) and 6-month follow-up (*P* < 0.05). | ALT: intervention group better than control group (*P* < 0.05) at 3-month, but NS observed at 6-month follow-up.  NS in AST.  Adverse events: 15 cases “flu-like” symptoms in control group; 3 cases severe diarrhea in intervention group (1 stop treatment). | 6 months | Good  (Modified Cochrane risk of bias 8/16) |
| Huang 2009 [25] | 83 (43/40) | ‘Fufang Biejia Ruangan Tablets’ (standardized prescription; oral 4 tablets, TID) and access to Adefovir dipivoxil 10 mg (oral, QD). | Adefovir dipivoxil 10 mg (oral, QD). | 1 year |  | NS in ALT & AST. | None | Poor  (Modified Cochrane risk of bias 6/16) |
| Kuang 2005 [26] | 53 (27/26) | Bie Jia Jian Decoction (standardized prescription; oral, one dose per day). | LVD 100 mg (QD). | 60 days | HA: intervention group better than control group (*P* < 0.05).  NS in PC-III & IV-C. | Improvement of related clinical symptoms: intervention group better than control group (*P* < 0.05).  NS in ALT & AST. | None | Good  (Modified Cochrane risk of bias 8/16) |
| Li 2006 [27] | 60 (30/30) | ‘Xiexian Oral Liquid’ (standardized prescription; oral 30 mL, QD), plus conventional care. | Placebo (physiological saline plus food coloring), plus conventional care. | 4 months | HA, LN & IV-C: intervention group better than control group (*P* < 0.01). | AST & improvement of related clinical symptoms: intervention group better than control group (*P* < 0.01).  NS in ALT.  Adverse events: 4 cases in intervention group (1 allergic, 3 diarrhea). | 6 months | Good  (Modified Cochrane risk of bias 8/16) |
| Li 2011 [28] | 88 (44/44) | ‘Anluo Huaxian Pills’ (standardized prescription; oral 6g, BID) and access to Adefovir dipivoxil 10 mg (oral, QD). | Adefovir dipivoxil 10 mg (oral, QD). | 9 months | HA, LN, PC-III & IV-C: intervention group better than control group (*P* < 0.05). | NS in ALT & AST.  Adverse events: none. | None | Poor  (Modified Cochrane risk of bias 5/16) |
| Lu 2010 [29] | 82 (42/40) | ‘Fufang Biejia Ruangan Tablets’ (standardized prescription; oral 4 tablets, TID) and access to Adefovir dipivoxil 10 mg (oral, QD). | Adefovir dipivoxil 10 mg (oral, QD). | 1 year | HA & LN: intervention group better than control group (*P* < 0.01).  NS in PC-III & IV-C. | ALT, AST & Improvement of related clinical symptoms: intervention group better than control group (*P* < 0.05).  Adverse events: none. | None | Good  (Modified Cochrane risk of bias 8/16) |
| Shen 2003 [33] | 68 (31/37) | ‘Ganxian Prescription’ (semi-standardized prescription, one dose per day) and access to LVD 100 mg (oral, QD). | LVD 100 mg (oral, QD). | 1 year | HA: intervention group better than control group (*P* < 0.05).  NS in LN & IV-C. | ALT: intervention group better than control (*P* < 0.05).  NS in AST.  Adverse events: none. | None | Good  (Modified Cochrane risk of bias 8/16) |
| Shen 2005 [34] | 120 (40/40/40) | Group I: ‘Ganxian Recipe’ (semi-standardized prescription, one dose per day).  Group II: ‘Ganxian Recipe’ (same as Group I) and access to LVD 100 mg (oral, QD). | LVD 100 mg (oral, QD). | 2 years | HA, LN & IV-C: Group II better than control group (*P* < 0.05).  NS in HA, LN & IV-C comparing Group I with control group. | ALT: Group II better than control group (*P* < 0.05), NS comparing Group I with control group.  NS in AST.  Adverse events: 8 cases diarrhea (3 in Control group, 5 in Group I), 2 cases dizziness in Group II, 3 cases upper respiratory infection-like symptoms in control group. | None | Poor  (Modified Cochrane risk of bias 7/16) |
| Sun 2010 [35] | 55 (30/25) | ‘Anluo Huaxian Pills’ (standardized prescription; oral 6g, TID) and access to Adefovir dipivoxil 10 mg (oral, QD). | Adefovir dipivoxil 10 mg (oral, QD). | 48 weeks | HA: intervention group better than control group (*P* < 0.05).  NS in LN, PC-III & IV-C. | ALT, AST & Improvement of related clinical symptoms: intervention group better than control group (*P* < 0.05). | None | Poor  (Modified Cochrane risk of bias 7/16) |
| Wang 2006 [36] | 160 (50/60/50) | Group I: experienced clinical decoction (semi-standardized prescription, one dose per day).  Group II: Group I plus control group. | LVD 100 mg (oral, QD). | 6 months | HA, LN, PC-III & IV-C: Group II better than control group at 3 and 6 months (*P* < 0.01); Group I better than control group in HA at 3 and 6 months (*P* < 0.01). |  | None | Good  (Modified Cochrane risk of bias 10/16) |
| Wang 2010 [37] | 98 (49/49) | ‘Fufang Biejia Ruangan Tablets’ (standardized prescription; oral 4 tablets, TID) and access to Adefovir dipivoxil 10 mg (oral, QD). | Adefovir dipivoxil 10 mg (oral, QD). | 1 year | HA, LN, PC-III & IV-C: intervention group better than control group (*P* < 0.05). | ALT: intervention group better than control group (*P* < 0.05). | None | Poor  (Modified Cochrane risk of bias 6/16) |
| Wei 2010 [38] | 44 (22/22) | ‘Fufang Biejia Ruangan Tablets’ (standardized prescription; oral 4 tablets, TID) and access to Adefovir dipivoxil 10 mg (oral, QD). | Adefovir dipivoxil 10 mg (oral, QD). | 1 year | HA, LN, PC-III & IV-C: intervention group better than control group (*P* < 0.05). | NS in ALT & AST. | None | Good  (Modified Cochrane risk of bias 9/16) |
| Xie 2009 [39] | 62 (32/30) | ‘Huaxian Fugan Prescription’ (standardized prescription; oral 100 mL, BID), plus conventional care. | Conventional care. | 6 months | HA, LN, PC-III & IV-C: intervention group better than control group (*P* < 0.05). | ALT, AST & Improvement of related clinical symptoms: intervention group better than control group (*P* < 0.01). | None | Good  (Modified Cochrane risk of bias 8/16) |
| Yang 2009 [40] | 120 (60/60) | ‘Fufang Biejia Ruangan Tablets’ (standardized prescription; oral 4 tablets, TID), and access to LVD 100 mg (oral, QD) plus conventional care. | LVD 100 mg (oral, QD) plus conventional care. | 6 months | HA, LN, PC-III & IV-C: intervention group better than control group (*P* < 0.05). | NS in ALT & AST. | None | Good  (Modified Cochrane risk of bias 9/16) |
| Yin 2004 [41] | 102 (52/50) | ‘Herbal Compound 861’ (standardized prescription; oral 7 capsules, TID). | Placebo (shape consistent with ‘Herbal Compound 861’; oral 7 capsules, TID). | 24 weeks | PC-III, MMP9, TIMP1 & TIMP2: intervention group better than control group (*P* < 0.05).  NS in HA, LN, IV-C, MMP1 & MMP2. | ALT & AST: intervention group better than control group (*P* < 0.05).  NS in Improvement of related clinical symptoms.  Adverse events: dry throat, nausea, abdominal discomfort, constipation in both groups (the number of patients not mentioned). | None | Good  (Modified Cochrane risk of bias 8/16) |
| Zhang 2000 [42] | 78 (39/39) | ‘Kanggan Xianfang’ (standardized prescription; oral 8g, TID) plus conventional care. | Conventional care. | 6 months | HA, PC-III & TGF-β1: intervention group better than control group (*P* < 0.05). | ALT & Improvement of related clinical symptoms: intervention group better than control group (*P* < 0.05). | None | Good  (Modified Cochrane risk of bias 8/16) |
