# Supplementary material for: Effectiveness of Chinese herbal medicine in treating liver fibrosis: a systematic review and meta-analysis of randomized controlled trials
Source: Chin Med. 2012 Feb 29;7:5. doi: 10.1186/1749-8546-7-5 (PMC3310806; doi:10.1186/1749-8546-7-5)
Supplement: Additional file 2 — Funnel plots (with pseudo 95% CI) for the primary outcomes (including HA, LN, PC-III and IV-C) of the included studies in the meta-analysis. (A) HA. (B) LN. (C) PC-III. (D) IV-C. [file 1749-8546-7-5-S2.PDF]

Additional file 2. Funnel plot (with pseudo 95% CI) for the primary outcomes of the included studies in the meta-analysis. MD: mean difference; SE: standard error. (A) HA. (B) LN. (C) PC-III. (D) IV-C.

A

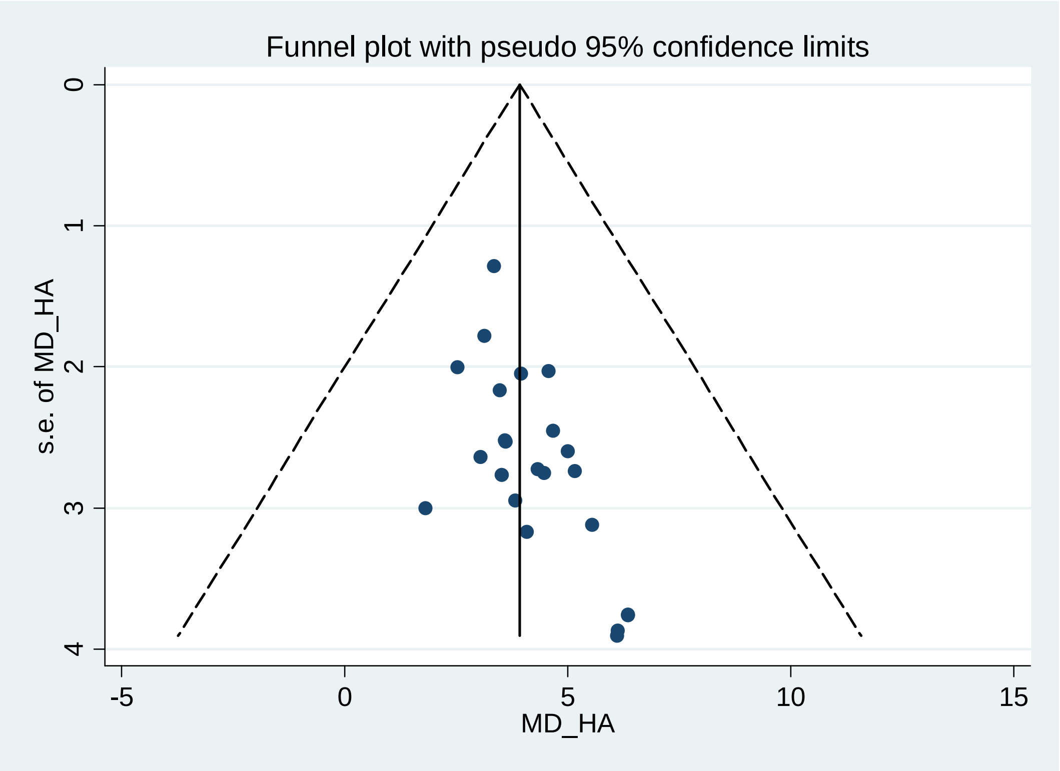

B

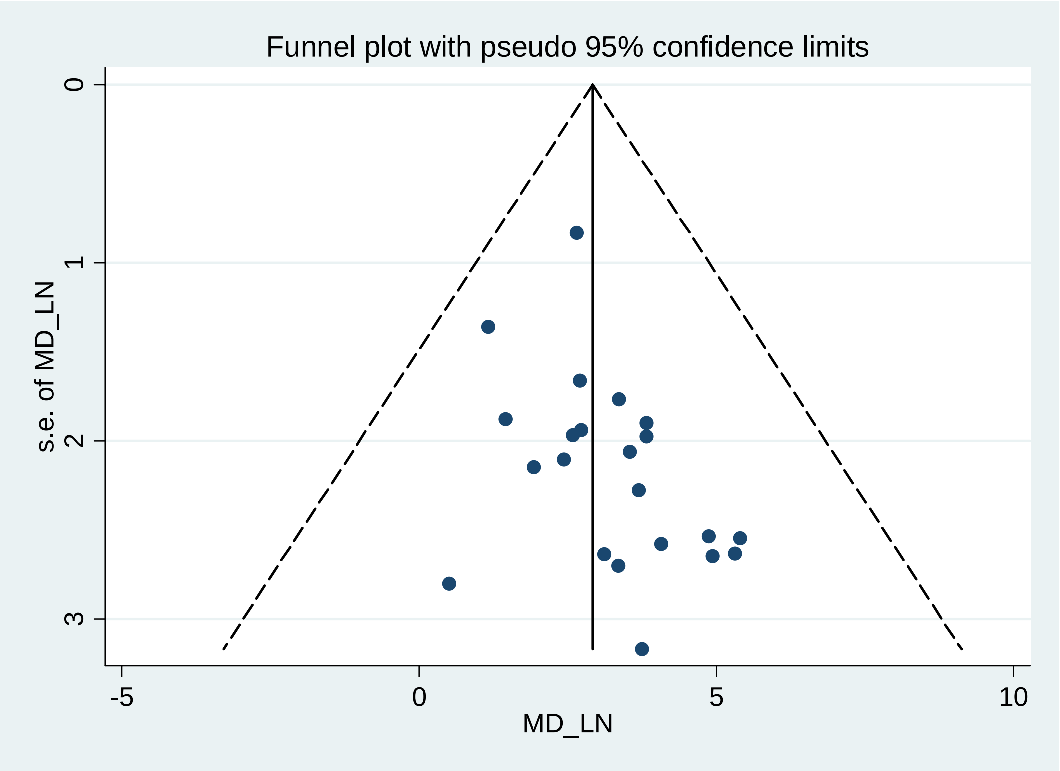

C

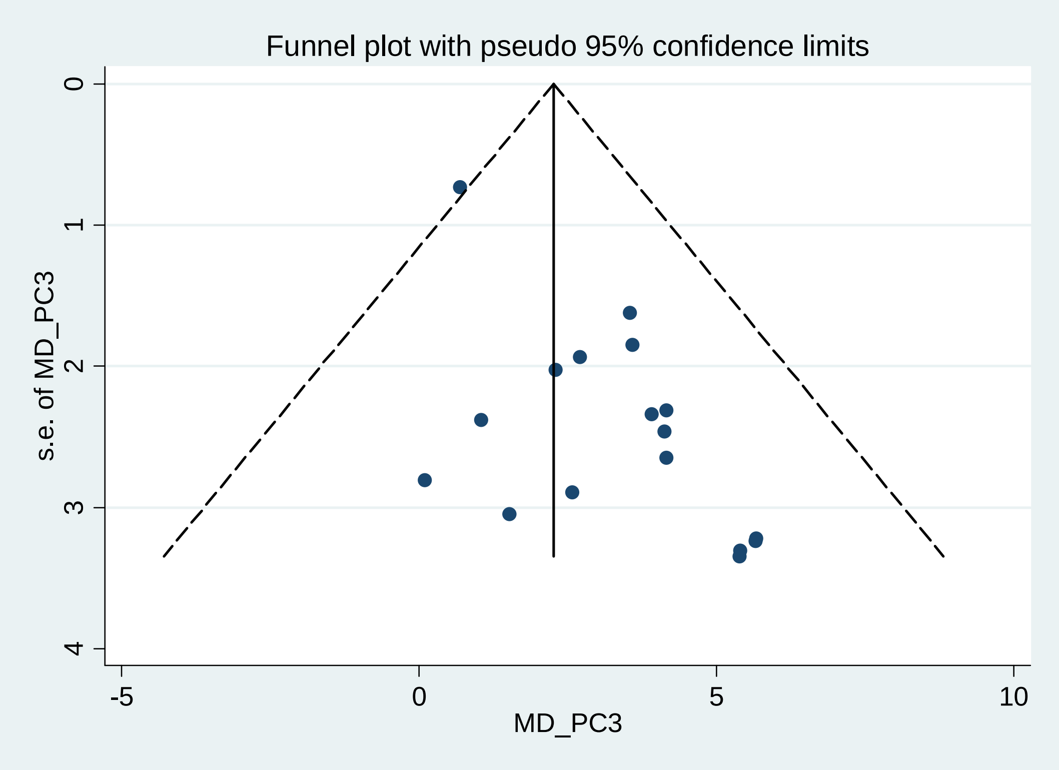

Funnel plot with pseudo 95% confidence limits
s.e. of MD_PC3
0
1
2
3
4
-5
0
5
10
MD_PC3

D

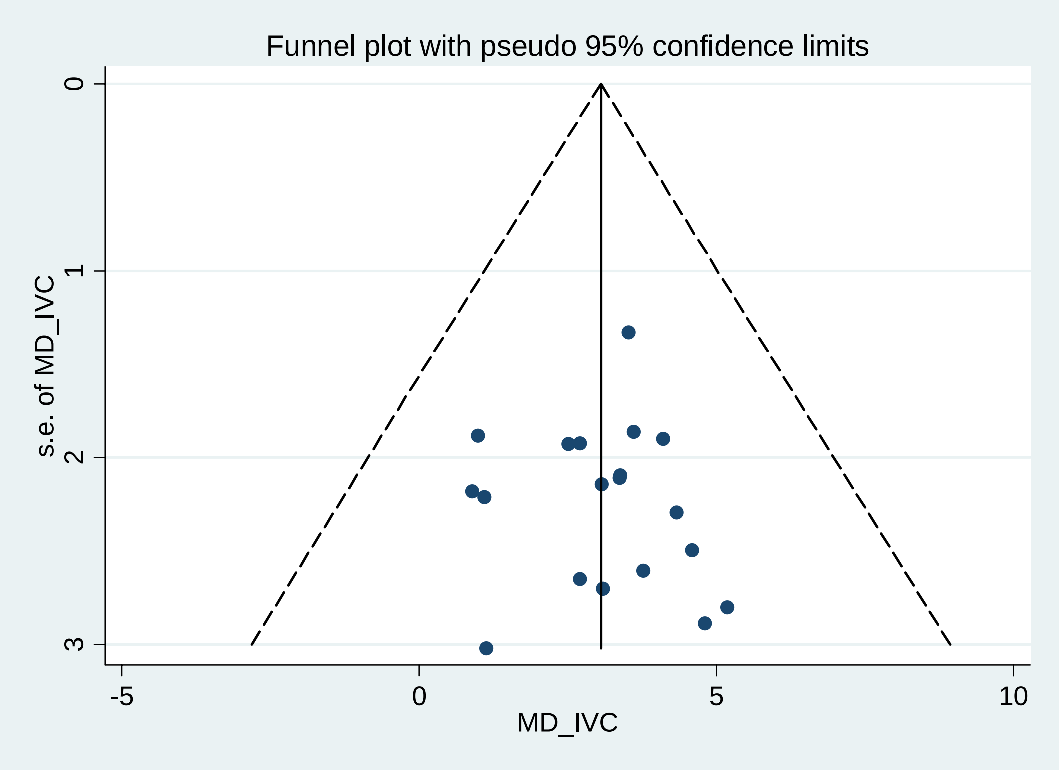

Funnel plot with pseudo 95% confidence limits
s.e. of MD_IVC
0
1
2
3
-5
0
5
10
MD_IVC
